# Integrated untargeted/targeted metabolomics identifies a putative oxylipin signature in patients with atrial fibrillation and coronary heart disease

**DOI:** 10.1515/jtim-2023-0141

**Published:** 2024-11-06

**Authors:** Lei Li, Yingyuan Lu, Zhiyong Du, Meng Fang, Ying Wei, Wenxin Zhang, Yisheng Xu, Jiaxu Sun, Xiangrui Zeng, Guomin Hu, Lingli Wang, Yong Jiang, Shuwang Liu, Yida Tang, Haiyi Yu, Pengfei Tu, Xiaoyu Guo

**Affiliations:** Department of Cardiology, Peking University Third Hospital; State Key Laboratory of Vascular Homeostasis and Remodeling, Peking University; NHC Key Laboratory of Cardiovascular Molecular Biology and Regulatory Peptides, Peking University; Beijing Key Laboratory of Cardiovascular Receptors Research, Beijing 100191, China; State Key Laboratory of Natural and Biomimetic Drugs, School of Pharmaceutical Sciences, Peking University, Beijing 100191, China; The Key Laboratory of Remodeling-Related Cardiovascular Diseases, Ministry of Education, National Clinical Research Center for Cardiovascular Diseases, Beijing Anzhen Hospital, Capital Medical University, Beijing 100029, China; Beijing Institute of Heart Lung and Blood Vessel Disease, Beijing 100029, China; Waters Technologies Ltd., Beijing 102600, China

**Keywords:** atrial fibrillation, coronary heart disease, metabolomics, glycerophospholipid, oxylipin

## Abstract

**Background and Objective:**

Atrial fibrillation (AF) and coronary heart disease (CHD) are closely related to metabolic dysregulation. However, the metabolic characteristics of AF patients with concomitant CHD remain unclear. The aims of this study were to elucidate the metabolic profiles of patients with AF and CHD to seek new therapeutic targets and related factors of AF combined with CHD.

**Methods:**

Untargeted metabolomics and targeted oxylipins profiling were performed to characterize the serum metabolome landscape of patients with AF, CHD, and AF comorbid CHD.

**Results:**

The serum metabolic fingerprints of patients with AF comorbid CHD were significantly differentiated from normal controls (NC) and individuals with AF or CHD alone, and the differentiated metabolites dominated by a variety of lipid alterations in the phospholipid and fatty acid metabolism. Furthermore, the targeted profiles of oxylipins demonstrated that the levels of arachidonic acid derivatives including prostaglandins, leukotrienes, hydroxy-docosahexaenoic acids, hydroxy-eicostetraenoic acids and hydroxy-eicosatrienoic acids in patients with AF and CHD were significantly different from those in the NC, AF, and CHD groups. Several prostaglandins were positively associated with echocardiographic indicators of myocardial remodeling.

**Conclusions:**

This study updates metabolic insights of AF and CHD and provides potential therapeutic targets for preventing or treating AF comorbid CHD.

## Introduction

The prevalence of atrial fibrillation (AF), the most common persistent arrhythmia around the world, is still on the increase.^[1]^ Heart failure and stroke are the primary causes of disability and death in AF.^[2,3]^ The clinical presentation of AF is complex and can often be accompanied by a variety of disease conditions. Equally important as a health condition with high morbidity and mortality worldwide is coronary heart disease (CHD), a condition in which a coronary artery stenosis or occlusion event resulting from atherosclerotic plaque leads to heart failure.^[4]^ It has been found that AF and CHD can mutually affect the development and progression of each of these conditions. For example, AF can contribute to the progression of CHD through mechanisms involving atherosclerosis, mismatch of blood supply and oxygen consumption, and thrombosis. On the other hand, CHD can exacerbate AF by affecting reentry formation, focal ectopic activity, and neural remodeling.^[5]^ Clinical epidemiology investigations have shown that 10%–25% of patients with CHD are accompanied by AF, and 30%–40% of patients with AF also suffer from CHD.^[6,7,8]^ Despite this degree of clinically diagnosed co-morbidity, there remains a lack of understanding of the molecular features of AF, CHD or the co-morbid condition. of the metabolite profile of patients with AF combined with CHD compared to those with CHD or AF alone.

Metabolomics has merged to become a highly specific and useful tool for disease diagnosis and drug efficacy evaluation, especially in the discovery of metabolic pathways of various diseases.^[9]^ In previous studies, targeted and untargeted metabolomics were reportedly carried out with samples from blood, urine, and even from tissue samples of patients with cardiovascular diseases and related model animals.^[10,11]^ Several studies have described a series of metabolites related to cardiovascular diseases such as myocardial infarction,^[12]^ heart failure,^[13,14]^ AF^[15]^ and diabete^[16]^. These metabolic markers were related to amino acid metabolism, lipid metabolism, and purine metabolism. The discovery of new cardiovascular disease related metabolites remains an area of intense research interest.^[17]^

Recent studies have shown that cardiovascular disease is inextricably associated with the metabolism of polyunsaturated fatty acids (PUFAs).^[18,20]^ Oxylipins are biologically active lipids produced by PUFAs through three biochemical pathways: namely cyclooxygenase (COX), lipoxygenase (LOX), and CYP450.^[21]^ The immunological functions of oxylipins include the regulation of inflammation, apoptosis, cell proliferation, blood coagulation, vascular permeability, and blood pressure.^[22]^ The oxylipins synthesized by the COX pathway are mostly prostaglandins and thromboxanes, which are involved in the regulation of blood pressure, platelet aggregation, inflammation, and stimulation of smooth muscle contraction.^[23]^ On the other hand, hydroxy-eicosatetraenoic acids (HETEs) are produced by the LOX pathway. Increased HETEs were found in essential hypertension, indicating that it may be involved in the pathogenesis of hypertension.^[24]^ 12-HETE can induce cell hypertrophy and fibrosis,^[25]^ whereas 15-HETE is related to heart failure caused by myocardial fibrosis.^[26]^ Epoxyeicosatrienoic acids (EETs) are produced by CYP enzyme metabolism and have shown anti-inflammatory effects, regulation of vasodilation, promotion of angiogenesis, and mitochondrial protection in cardiovascular diseases.^[27]^ In an ischemic model, EETs mediated mitochondrial protective effects, and could improve cardiac function.^[28,29,30,31]^ Finally, secondary preventive drugs for CHD, such as aspirin, statins, and anticoagulants used in patients with AF, are closely related to the oxylipins metabolic pathway.^[32-33]^

In this study, we performed both an untargeted and a targeted metabolomic assessment of human serum samples from normal controls (NC), patients with AF, with CHD, and in patients with both AF and CHD. From these data, we identified putative metabolite signatures that may be informative to distinguish between each of the four groups. Considering the influence of medication on metabolic pathways in patients with AF and CHD, we analyzed the relationship between anticoagulant, aspirin and statins and the level of metabolites. We also sought to explore the potential associations of metabolite signatures with myocardial remodeling-related echocardiographic markers.

## Methods

### Patient enrolment

This is a single-center, prospective observational cohort study, and the workflow is shown in Figure S1. From October 2018 to October 2019, we conducted the AF cohort study by consecutively enrolling patients with AF without CHD, CHD without AF, and both AF and CHD who were admitted to the Cardiology Department of Peking University Third Hospital. We also recruited healthy controls who did not have AF or CHD, and who underwent physical examinations at Peking University Third Hospital during the same period. AF was confirmed by electrocardiogram/Holter and CHD was identified by coronary arteriography (CAG). Heart failure classification in our study was based on the clinical diagnosis provided by the attending physicians, in accordance with guidelines such as those from the American Heart Association/American College of Cardiology.^[34]^ According to the criteria below, we screened out 159 patients from the AF cohort. The exclusion criteria included age less than 18 years old, congenital heart disease, valvular heart disease, severe heart failure (NYHA ≥ III class), abnormal liver function (aspartate aminotransferase or alanine aminotransferase above the upper limit of normal), severe kidney disorders (creatinine > 354 μmol/L), current infection, hematological disease, thyroid-related hospital diagnoses, and those with no signed informed consent.

This study protocol was conducted in accordance with the Declaration of Helsinki and approved by the Ethics Review Boards of Peking University Third Hospital (Approval number: 077-02, Beijing, China). Written informed consent was obtained from all participants.

### Measurement of clinical data

Demographic and clinical information of all participants was collected by experienced physicians, including age, sex, and previous medical history. Venous blood samples were obtained from subjects following overnight fasting (8 hours), and samples once collected were stored at 4°C. Creatinine (Cr) was immediately measured by clinical laboratories in Peking University Third Hospital using standard laboratory procedures. Other blood samples were processed within 30 minutes after collection by centrifugation at 3000 ×*g* for 15 minutes at 4°C. To avoid repeated freeze–thaw cycles, each sample was divided into 0.2 mL aliquots and frozen immediately at –80°C. Two-dimensional transthoracic echocardiography was performed, using the GE Vivid E9 system and a 3.5 MHz transducer. Standard views including M-mode, 2D images, and Doppler and color-Doppler data were acquired from the parasternal and apical views (4-, 2-, and 3-chamber view), and parameters recorded.

### Materials and chemicals

Acetonitrile, methanol, methyl tert-butyl ether (MTBE), and formic acid of LC-MS grade were all purchased from Fisher Scientific (Pittsburg, USA). Ultrapure water was produced from a Millipore-Q system (Millipore, MA, USA). Leucine enkephalin, L-leucine-5, 5, 5-d_3_, L-phenyl-d_5_-alanine, and stearic acid-18, 18, 18-d_3_ were purchased from Sigma-Aldrich (St. Louis, MO, United States). Lysophosphatidylcholine (LysoPC) (19:0), and phosphatidylcholine (PC) (17:0) were purchased from Avanti Polar Lipids, Inc. (Alabaster, United States). 11,12-EET-d_11_, 14,15-EET-d_11_, 8,9-EET-d_11_, 12,13-diHOME-d_4_, 13-HODE-d_4_, 15d PGJ2-d_4_, 6k PGF1α-d_4_, 8-iso PGF2αVI-d_4_, 9,10-diHOME-d_4_, 9-HODE-d_4_, dhk PGF2α-d_4_, LTB4-d_4_, PGD2-d_4_, PGE2-d_4_, Resolvin E1-d_4_, TXB2-d_4_, 12-HETE-d_8_, 15-HETE-d_8_, 5-HETE-d_8_, Arachidonic acid-d_8_ were supplied from Cayman Chemical (Ann Arbor, Michigan, United States). All reagents were of analytical grade as a minimum.

### Serum sample pretreatment for untargeted metabolomics

Frozen serum samples were thawed at 4°C, and 50 μL of each sample was precipitated by adding 200 μL of ice-cold methanol-water (7:3, v/v) containing 2 μg/mL L-leucine-5, 5, 5-d_3_, L-phenyl-d_5_-alanine, stearic acid-18, 18, 18-d_3_, PC 17:0/17:0, and LysoPC (19:0) as the internal standard (IS). The mixture was vortex–mixed for 10 minutes, sonicated for 10 minutes in an ice-water bath, vortex–mixed for 3 minutes again, and then centrifuged at 21130 ×*g* for 10 minutes at 4°C.

Subsequently, 200 μL of the supernatant was lyophilized under reduced pressure at 20°C. The residue was reconstituted in 200 μL of methanol/water (50/50, v/v) and then centrifuged at 21130 ×*g* for 10 min before UPLC-TOF/MS analysis.

### UPLC-Q-TOF/MS analysis for untargeted metabolomics

A Waters Xevo G2-XS QTOF system (Waters Corp., Milford, MA, USA) via an electrospray ionization (ESI) interface was used for this analysis. An ACQUITY UPLC HSS T3 column (100 mm × 2.1 mm, 1.8 μm) was used for separation. The mobile phase consisted of acetonitrile with 0.1% formic acid (v/v) and ultrapure water with 0.1% formic acid (v/v). Gradient elution conditions were as follows: 0–1.5 minutes, 0%–5% B; 1.5–3.5 minutes, 5%–35% B; 3.5–4.5 minutes, 35%–50% B; 4.5–5.5 minutes, 50%–70% B; 5.5–9.0 minutes, 70%–100% B; 9.0–11.5 minutes, 100% B; 11.5–13.5 minutes, 100%–0% B; 13.5 – 16.5 minutes, 0% B. The flow rate was 0.4 mL/min and the oven temperature was 40°C. The injection volume of samples was 1 μL for positive ion mode and 3 μL for negative ion mode.

The ion source parameters of MS were kept as follows: capillary voltage: 2.5 kV; cone voltage: positive mode (ESI+) 40 V and negative mode (ESI–) 35 V; source temperature: 110°C; desolvation gas temperature: positive mode (ESI+) 400°C and negative mode (ESI–) 450°C; desolvation gas flow: 700 L/h; cone gas rate: 50 L/h; collision energy (CE): 10–60 eV; Scanning range of mass spectrometry: 50-1100 Da. Data was acquired using a LockSpray interface and leucine enkephalin was used as the reference compound with m/z 556.2771 for ESI+ and 554.2615 for ESI–.

### Plasma sample pretreatment for targeted metabolomics

For each sample, a 50 μL aliquot was thawed at 4°C and then added to 200 μL of ice-cold methanol with 1% formic acid (v/v) containing the internal standard (a mixture of deuterated lipid oxide standard). The mixture was vortexed and mixed for 2 min, incubated on ice for 3 min, and centrifuged at 21130 ×*g* for 15 min at 4°C. Subsequently, the upper phase was transferred to a clean centrifuge tube. Meanwhile, the precipitate was mixed with 200 μL of ice-cold MTBE/methanol (7:3, v/v) with 1% formic acid. After vortex-blending for 2 min and incubation on ice for 3 min, the mixture was centrifuged at 21130 ×*g* for 15 min at 4°C. The upper phase was combined and lyophilized under reduced pressure at 20°C. To reconstitute the residue, 80 μL of methanol-acetonitrile (1:1, v/v) was used, and the sample centrifuged at 21130 ×*g* for 10 min before UPLC/MS analysis.

### UPLC-Qtrap/MS analysis for targeted metabolomics

The analysis was obtained on a Waters ACQUITY UPLC system coupled with an AB SCIEX Qtrap 4500 mass spectrometer (SCIEX, Foster, USA) via an ESI interface. The UPLC system consisted of a binary solvent delivery system, an autosampler, a column compartment, and a diode-array detector. Chromatographic separations were performed on an ACQUITY UPLC BEH C18 column (2.1 × 100 mm, 1.7 μm). The mobile phase, consisting of 0.1% formic acid in water + acetonitrile-isopropanol (9:1, v/v), was programmed in gradient as follows: 0–2 min, 25%–25% B; 2–10 min, 25%–95% B; 10–12 min, 95%–95% B; 12–15 min, 95%–25% B and the flow rate was 0.4 mL/min. The temperatures of the column oven and autosampler were maintained at 40°C and 4°C, respectively. All MSE data were acquired with the negative ion mode. Other mass parameters were set as follows: the nebulizer (GS1), 50 psi; heater (GS2), 50 psi; curtain (CUR), 35 psi; ion spray needle voltage, –4500 V; turbo gas temperature, 500°C; collisional activated dissociation (CAD) gas, medium level. Based on previous studies by our research group, the optimized MRM ion pair, collision energy (CE), and deionization potential (DP) of 20 deuterated lipid oxide standards and 99 oxylipins are shown in Table S1 and Table S2. The representative total ion chromatography chromatogram of oxylipins in serum samples is described in Figure S2.

### Data processing and statistical analysis

The matrix of LC/MS data was submitted for multivariate statistical analysis (MVA) by SIMCA-P software (v14.1, Umetric, Umeå, Sweden) and the quality of the MVA model was controlled by the validation parameters of R^2^ and Q^2^. Firstly, both the dynamic trajectory and the clustering trends of different groups were traced by unsupervised principal component analysis (PCA). Secondly, supervised orthogonal partial least squares discriminate analysis (OPLS-DA) was performed to identify significantly differential metabolites and the CV-ANOVA *P* value was calculated to test the reliability of the OPLS-DA model. The S-line coefficient-coded loading plot and the combination of variable importance plot (VIP ≥ 1.5) and S-plot were used to identify the differentially changed metabolites in different groups for LC/MS-based metabolomics. Finally, Student’s *t*-test (*P* < 0.05) and the univariate receiver operating characteristic (ROC) curve by using the area under the ROC curve (AUC ≥ 0.6) were both applied to assess the statistical accuracy of signals representing these metabolites. The heatmap cluster analysis and correlation coefficients for mapping metabolite alterations were built using TBtools (v. 66831) and MetaboAnalyst (http://www.metaboanalyst.ca/).

In Table 1, the Kolmogorov-Smirnov test was used to test normality and a *P* value > 0.05 was defined as normally distributed data. Data were expressed as the mean value ± standard deviation (X ± SD) for continuous variables of a normal distribution, median ± quartile ranges (QR) for non-normal distribution, and percentages for categorical variables as appropriate. We used multiple linear regressions to explore potential associations between oxylipins and echocardiographic data after adjustment for other confounders. Non-normal distributed variables were log transformed when put into the model. We analyzed the associations between the levels of oxylipins and binary factors by Wilcoxon test, and continuous variables by Spearman correlation. These analyses were carried out using RStudio version 4.1.2. Statistical significance was defined as a two-sided *P* value < 0.05 for all comparisons.

**Table 1:** Baseline clinical characteristics of subjects in this study

Baseline	Control (*N* = 38)	AF (*N* = 39)	CHD (*N* = 40)	AF + CHD (*N* = 42)
Age (years)	60.5 [55.0, 64.0]	64.0 [60.0, 71.0]	65.5 [60.8, 72.3]	66.0 [61.0, 73.0]
Female	13 (34.2%)	6 (15.4%)	6 (15.0%)	6 (14.3%)
BMI (kg/m^2^)	26.2 [24.2, 28.1]	24.7 [23.3, 26.2]	23.7 [23.6, 24.9]	26.3 [23.8, 27.7]
Smoking	12 (31.6%)	18 (46.2%)	20 (50.0%)	28 (66.7%)
Diabetes	0 (0%)	5 (12.8%)	16 (40.0%)	14 (33.3%)
Hypertension	5 (13.2%)	25 (64.1%)	31 (77.5%)	28 (66.7%)
Hyperlipidemia	6 (15.8%)	11 (28.2%)	22 (55.0%)	20 (47.6%)
Heart failure	0 (0%)	3 (7.7%)	0 (0%)	6 (14.3%)
Cr (*μ*mol/L)	75.5 [70.3, 84.3]	87.0 [79.0, 92]	84.0 [76.3, 88]	86.0 [77.5, 97]
LAD (mm)	33.6 [32.3, 34.7]	37.6 [35.5, 41.6]	36.2 [34.5, 38.1]	39.9 [35.4, 45.3]
LVEF (%)	69.5 [67.0, 72.0]	67.0 [65.0, 70.5]	69.5 [66.8, 72.3]	69.0 [64.0, 73.8]
LVEDD (mm)	46.8 (3.42)	48.7 (5.80)	47.4 (3.28)	49.2 (6.15)

AF: atrial fibrillation; CHD: coronary heart disease; BMI: body mass index; Cr: Creatinine; LAD: left atrial diameter; LVEF: left ventricular ejection fraction; LVEDD: left ventricular end-diastolic dimension.

## Results

### Baseline clinical characteristics

Participants were divided into four groups according to the diagnosis: 38 healthy controls, 39 patients with AF, 40 patients with CHD, and 42 patients with AF and CHD (Table 1). The four groups were named the NC group, AF group, CHD group, and AF + CHD group, respectively, and the workflow of this study was show in Figure S1. A total of 159 participants (128 males and 31 females, with a median age of 64 years old) were analyzed. The AF + CHD group comprised the largest proportions of smokers (66.7%) and heart failure (14.3%) compared with participants within the other three groups. The median creatinine level was 84 μmol/L. For echocardiographic data, measurements of left atrial diameter (LAD) and left ventricular end-diastolic dimension (LVEDD) were the largest for the AF + CHD group, while the control group had the smallest LAD and LVEDD measurements. The differences in LVEF across the four groups did not appear to be dramatic.

### Metabolic profiles of serum samples from the four cohorts

**Figure 1:**
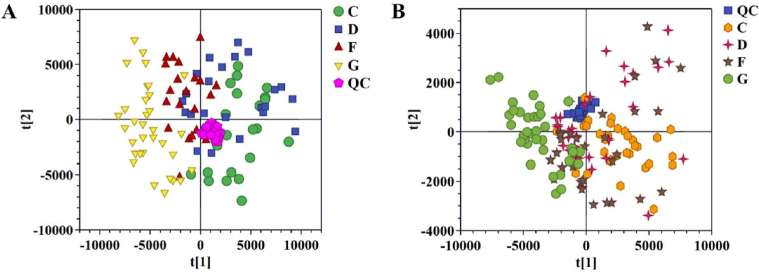
Pattern analysis of data from the metabolic profiles of serum by UPLC-Q-TOF/MS. A. PCA scores plot of the serum samples in positive mode. B. PCA scores plot of the serum samples in negative mode. PCA: principal component analysis; NC: normal control; AF: Atrial fibrillation; CHD: coronary heart disease. C: NC group; D: AF + CHD group; F: AF group; G: CHD group.

After the raw MS data was processed, PCA based-dynamic trajectory analysis was constructed to reveal the metabolite expression profiles of the AF group, CHD group, AF + CHD group, and NC group in both positive (Figure 1A) and negative (Figure 1B) ion modes.

Quality control (QC) samples were tightly clustered in the results of PCA score plots. The R^2^X and Q^2^ of the PCA models were 0.837 and 0.251 for positive ion mode, 0.848 and 0.330 for negative ion mode. The spots of the NC group were clustered separately from the spots of AF group, CHD group, and AF + CHD group, implying distinct metabolic profiles between health and disease status. The spots of the AF group were completely separated from that of the CHD group, but overlapped with those of the AF + CHD group, indicating that the metabolic profiles of AF and CHD were different, and the metabolic profile of AF combined with CHD was more similar to that of AF.

**Figure 2:**
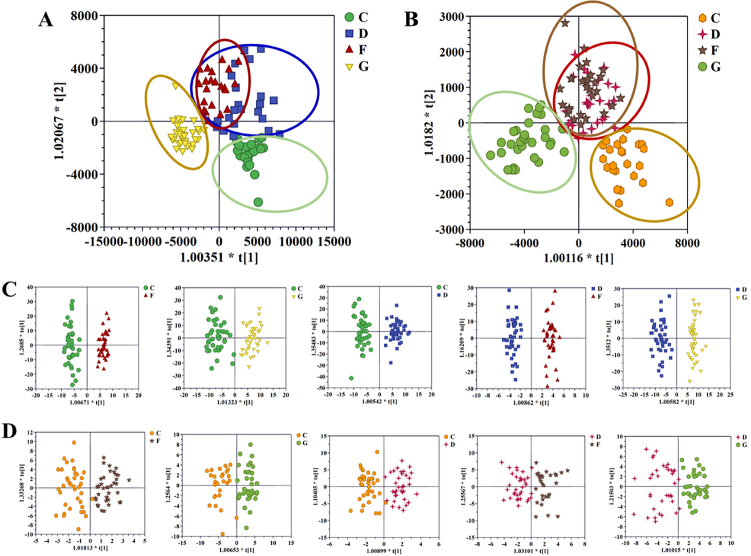
OPLS-DA score plot of the four cohorts in positive mode (A), the four cohorts in negative mode (B), C and F patients, C and G patients, C and D patients, D and F patients and D and G patients in the positive mode (C), and in the negative mode (D). NC: normal control; AF: atrial fibrillation; CHD: coronary heart disease. C: NC group; D: AF + CHD group; F: AF group; G: CHD group.

Next, supervised OPLS score plots were established to further select differentially expressed ions. The results of comparing the NC to AF group, NC to CHD group, NC to AF + CHD group, and AF to AF + CHD group are shown in Figure 2, and all OPLS score plots could be clearly distinguished in positive and negative ion modes. The evaluation parameters (R^2^X, R^2^Y, Q^2^, and CV-ANOVA *P* value) of OPLS met the statistical requirements as follows: OPLS analysis parameters in positive ion mode: NC versus AF, R^2^X = 0.324, R^2^Y = 0.961, Q^2^ = 0.766, CV-ANOVA *P* value = 1.3091e^-18^; NC versus CHD, R^2^X = 0.395, R^2^Y = 0.88, Q^2^ = 0.406, CV-ANOVA *P* value = 6.3254e^-6^; NC versus AF + CHD, R^2^X = 0.427, R^2^Y = 0.927, Q^2^ = 0.551, CV-ANOVA *P* value = 2.7913e^-9^; AF and AF + CHD, R^2^X = 0.414, R^2^Y = 0.962, Q^2^ = 0.302, CV-ANOVA *P* value = 0.0079; CHD and AF + CHD, R^2^X = 0.368, R^2^Y = 0.935, Q^2^ = 0.702, CV-ANOVA *P* value = 1.2945e^-16^. OPLS analysis parameters in negative ion mode: NC versus AF, R^2^X = 0.391, R^2^Y = 0.816, Q^2^ = 0.51, CV-ANOVA *P* value = 4.4684e^-8^; NC versus CHD, R^2^X = 0.336, R^2^Y = 0.838, Q^2^ = 0.723, CV-ANOVA *P* value = 4.4257e^-16^; NC versus AF + CHD, R^2^X = 0.386, R^2^Y = 0.843, Q^2^ = 0.524, CV-ANOVA *P* value = 9.4711e^-8^; AF and AF + CHD, R^2^X = 0.245, R^2^Y = 0.748, Q^2^ = 0.585, CV-ANOVA *P* value = 1.0178e^-10^; CHD and AF + CHD, R^2^X = 0.309, R^2^Y = 0.762, Q^2^ = 0.648, CV-ANOVA *P* value = 1.4356e^-14^. The above results showed that the established OPLS model had prediction ability and high reliability.

### Differential metabolites and pathways for each of the cohorts

**Figure 3:**
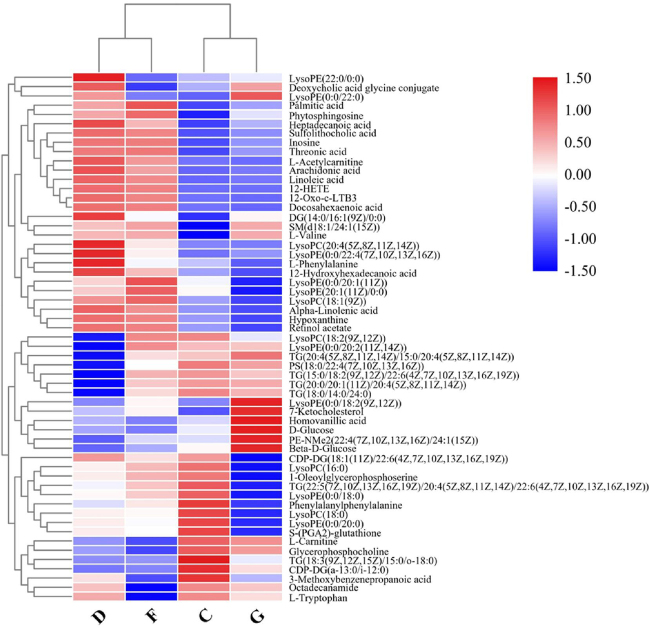
Heatmap of differential metabolite intensities in different groups. NC: normal control; AF: atrial fibrillation; CHD: coronary heart disease. C: NC group; D: AF + CHD group; F: AF group; G: CHD group.

A heatmap of the individual levels of significantly altered serum metabolites (*P* < 0.05) is shown in Figure 3. There were 36 differential metabolites between the NC and AF + CHD groups. Compared to the NC group, LysoPC(16:0), LysoPC(18:0), LysoPC(18:2(9Z,12Z)), TG(20:4(8Z,11Z,14Z,17Z)/22:5 (4Z,7Z,10Z,13Z, 16Z)/22:6(4Z,7Z,10Z,13Z,16Z,19Z)), S-(PG A2)-glutathione, LysoPE(0:0/20:0), LysoPE(0:0/18:0) TG (20:4(5Z,8Z,11Z,14Z)/15:0/20:4 (5Z,8Z,11Z,14Z)), TG(18:0/14:0/24:0), TG(16:0/20:4(5Z,8Z,11Z,14 Z)/24:1(15Z)), TG(18:3 (9Z,12Z,15Z)/15:0/o-18:0), PS(18:0/22:4(7Z, 10Z,13Z,16Z)), D-Glucose, and L-Tryptophan were significantly decrease in the AF + CHD group, but 12-Oxo-c-LTB3, α-linolenic acid, linoleic acid, arachidonic acid, 12-HETE, L-valine, L-acetylcarnitine, hypoxanthine, threonic acid, palmitic acid, phytosphingosine, SM(d18:1/24:1(15Z)), inosine, 3-methoxybenzenepropanoic acid, LysoPE(0:0/22:4 (7Z,10Z,13Z,16Z)), LysoPC(20:4(5Z,8Z,11Z,14Z)), LysoPE(20:1(11Z)/0:0), 12-hydroxyhexadecanoic acid, DG(14:0/16:1(9Z)/0:0), LysoPC(18:1 (9Z)), and L-phenylalanine were significantly increase in the AF + CHD group.

**Figure 4:**
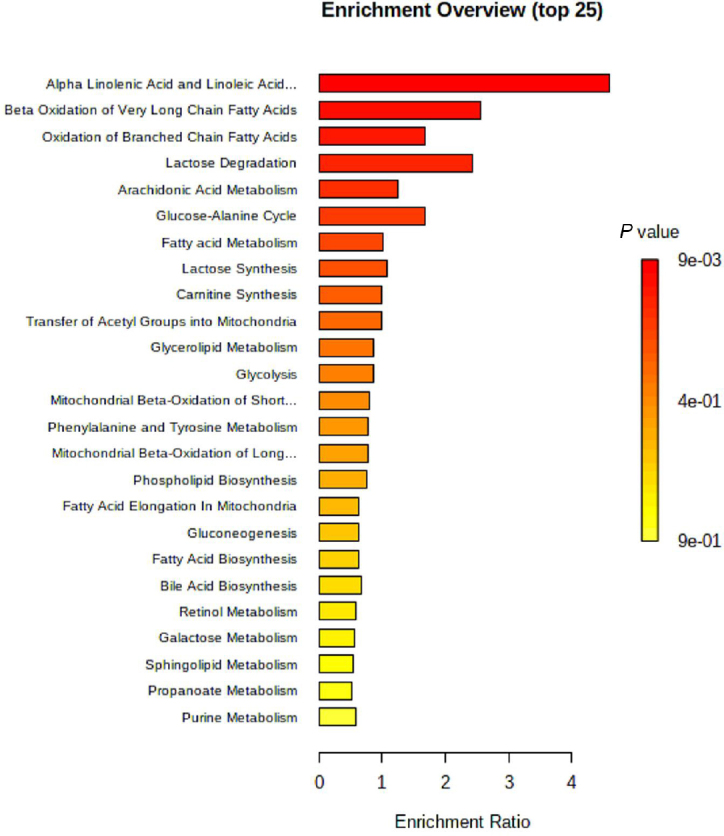
MetaboAnalyst based-enrichment analysis of the key metabolic alterations in the AF + CHD group. AF: atrial fibrillation, CHD: coronary heart disease.

From the differential analysis of metabolites across groups, it was evident that the annotated metabolite alterations in AF combined with CHD mainly included phospholipids, free fatty acids, and oxylipins. As shown in the heatmap visualization, there were significant differences in the metabolites between the NC group and the AF + CHD group. MetaboAnalyst analysis was used to analyze the metabolic pathways related to major differential metabolites. As shown in Figure 4, fatty acid metabolism and its downstream α-linolenic acid, linoleic acid, and arachidonic acid metabolism were mainly enriched with higher pathway impacts for these altered metabolites in patients with AF combined with CHD.

**Figure 5:**
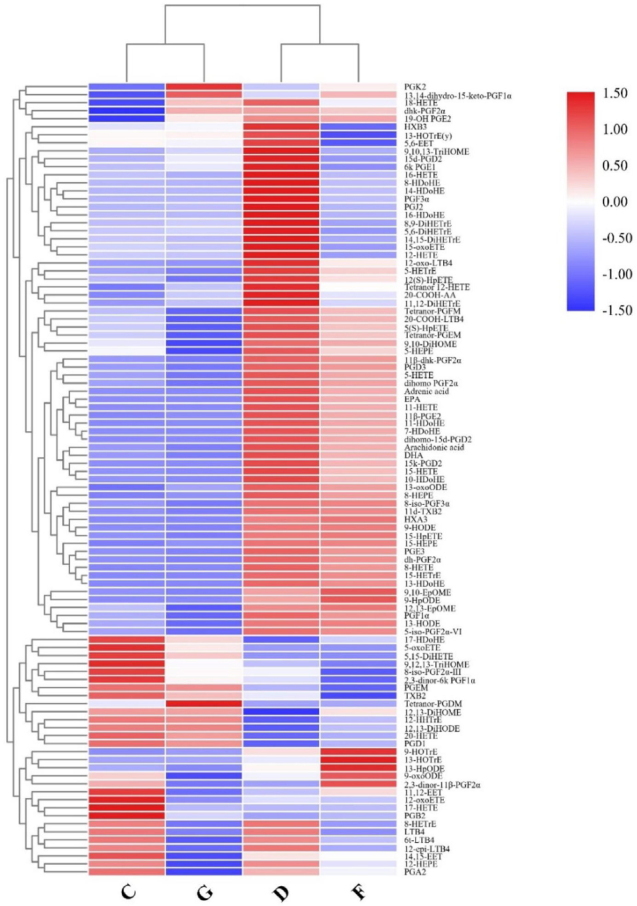
Heatmap of oxylipins intensities in different groups. NC: normal control, AF: atrial fibrillation, CHD: coronary heart disease. C: NC group; D: AF + CHD group; F: AF group; G: CHD group.

Targeted metabolic profiles of serum samples from the four cohortsWe performed targeted metabolic profiling to discover that oxylipins were most significantly altered in the metabolic profile from the AF + CHD group compared with the NC group. The serum levels of AA-derived oxylipins, including monohydroxides, hydroperoxides, epoxides, leukotrienes, and prostaglandins, in the AF co-morbid with CHD group were significantly different from those in the AF, CHD, or NC groups (Figure 5). The unique metabolites in the AF + CHD group were PGD1, 6-keto-PGE1, PGE3, PGF3α, PGJ2, 15-keto-PGD2, 12-oxo-LTB4, 16-HDoHE, 11-HETE, 12-HETE, 15-HETE, 12(S)-HpETE, tetranor 12-HETE, 14-HDoHE, 8-HDoHE, 15-HETrE, 5,15-diHETE, and 17-HDoHE. The significance of each these metabolites was further confirmed by univariate ROC curve analyses (Figure S3) and Student’s t-tests (*P* < 0.05). The combined ROC curve analysis for individual metabolic biomarkers of the AF + CHD group was showed in Figure S4 and the AUC values of the metabolites ranged from 0.732 to 0.976. These results suggest that oxylipins play an important role in patients with AF co-morbid with CHD.

### Correlation between candidate oxylipins, echocardiographic data and clinical risk factors

We further explored the potential association between candidate oxylipin species and echocardiographic data (LAD, LVEDD and LVEF) from all patients (Table 2). In the multiple models after adjustment for age, sex, smoking, BMI, diabetes, hypertension, hyperlipidemia, heart failure, AF, and CHD, we found that PGE3, PGJ2, and 15-keto-PGD2 were positively associated with LAD. In addition, 12(S)-HpETE was independently correlated with LVEDD after adjustment for other risk factors. The risk factors in the AF + CAD group were significantly higher than those in the other groups. We further analyzed the associations between the levels of oxylipins and several risk factors (including sex, smoking status, diabetes, hypertension, hyperlipidemia, and heart failure) in all subjects (Table S3). We found that oxylipins had no significant associations with these factors. No correlation was found between oxylipins and total cholesterol and LDLc levels in this study (Table S4).

**Table 2:** Regression analysis between the differential oxylipins and echocardiographic data

Oxylipins	LAD		LVEDD		LVEF	
		
	β (SE)	*P*	β (SE)	*P*	β (SE)	*P*
PGD1	0.002 (0.014)	0.91	0.23 (0.63)	0.71	-1.03 (0.81)	0.20
6-keto-PGE1	-0.015 (0.008)	0.09	-0.09 (0.38)	0.82	-0.53 (0.48)	0.27
PGE3	0.086 (0.034)	0.01*	-0.31 (1.52)	0.84	-1.51 (2.00)	0.45
PGF3α	0.004 (0.012)	0.71	0.25 (0.54)	0.64	0.81 (0.73)	0.27
PGJ2	0.043 (0.022)	0.05*	1.59 (0.93)	0.09	1.88 (1.26)	0.14
15-keto-PGD2	0.035 (0.017)	0.045*	0.71 (0.76)	0.35	0.32 (1.00)	0.75
12-oxo-LTB4	0.024 (0.022)	0.27	-0.87 (0.93)	0.35	-0.82 (1.22)	0.50
16-HDoHE	0.010 (0.008)	0.22	-0.03 (0.36)	0.93	-0.69 (0.46)	0.14
11-HETE	-0.001 (0.009)	0.91	-0.19 (0.41)	0.64	-0.34 (0.54)	0.53
12-HETE	0.004 (0.007)	0.59	0.09 (0.31)	0.77	-0.36 (0.41)	0.38
15-HETE	0.001 (0.012)	0.96	0.15 (0.51)	0.77	-1.17 (0.67)	0.08
12(S)-HpETE	0.001 (0.002)	0.93	-0.19 (0.09)	0.04*	0.02 (0.12)	0.89
tetranor 12-HETE	-0.002 (0.014)	0.91	-0.65 (0.57)	0.26	-0.59 (0.74)	0.43
14-HDoHE	0.014 (0.008)	0.08	-0.03 (0.34)	0.93	-0.73 (0.44)	0.10
8-HDoHE	0.003 (0.010)	0.79	-0.33 (0.44)	0.45	-1.11 (0.57)	0.05
15-HETrE	0.010 (0.014)	0.47	-0.23 (0.59)	0.69	-0.09 (0.77)	0.91
5,15-diHETE	0.000 (0.001)	0.51	-0.02 (0.03)	0.44	-0.05 (0.04)	0.18
17 HDoHE	-0.010 (0.016)	0.54	0.28 (0.68)	0.68	0.30 (0.89)	0.74

LAD: left atrial diameter; LVEDD: left ventricular end-diastolic dimension; LVEF: left ventricular ejection fraction; SE: standard error; *P < 0.05.

### Effect of medication on serum levels of oxylipins

We further explored the association between oxylipins and the use of aspirin, anticoagulants, or statins among all participants. We found that the level of PGD1, 12-oxo-LTB4, 12(S)-HpETE, and 15-HETrE were different between aspirin users and aspirin non-users (Table S5), and taking warfarin or direct oral anticoagulants had a significant impact on the levels of PGD1, PGE3, PGF3α, 12-oxo-LTB4, 11-HETE, 15-HETE, 12(S)-HpETE, tetranor 12-HETE, 8-HDoHE, 15-HETrE, 17 HDoHE (Table S6). The use of statins did not have a significant impact on the levels of oxylipins in this study (Table S7).

## Discussion

The study has provided a comprehensive metabolomics analysis of plasma samples from healthy controls, and from patients with AF, CHD, and AF + CHD. Our results we have found that the metabolic profiles of these four groups are different. The metabolic profile of the AF + CHD group is different from those of the AF group and CHD group, indicating that each condition is metabolically unique. Nevertheless, the metabolic pathway analysis of different metabolites showed that fatty acid metabolism, as well as α-linolenic acid and linoleic acid metabolism were the most significant metabolic pathways. Furthermore, we used targeted oxylipins analysis and found that the AF, CHD, and AF + CHD groups were significantly different in their metabolic profiles of oxylipins. The levels of some oxylipins were positively associated with echocardiographic indicators of myocardial remodeling. Our findings showed that unique oxylipin species in the serum of patients with AF + CHD may be informative as potential therapeutic targets for prevention or treatment of progression from AF or CHD, to a co-morbid disease.

Our results from untargeted metabolomics analyses showed that a variety of saturated fatty acids (SFAs), PUFAs, oxylipins, glycerophospholipids, triacylglycerol (TG), diacylglycerol (DG), and sphingomyelin (SM) components were altered in the serum of patients with AF co-morbid with CHD. Changes in lipid metabolites, consisting of phospholipids, sphingolipids, glycolipids, fatty acids, and acyl carnitine, are associated with pathological changes in cardiovascular disease.^[35]^ Previous studies have found that LysoPC and lysophosphatidylethanolamine (LysoPE) have multiple biological functions and participate in a variety of physiological and pathological processes. LysoPC is involved in cell membrane dissolution, apoptosis, and inflammation. LysoPC can induce reactive oxygen species (ROS) and apoptosis, and then affect the function of endothelial cells.^[36]^ LysoPE, considered to be important arrhythmogenic lipids, has multiple biological functions and participates in a variety of physiological and pathological processes.^[37]^ Phosphatidylserine (PS) has recently been widely demonstrated to have anti-inflammatory properties and is a messenger in apoptotic synapses.^[38]^ SM is involved in the pathogenesis of cardiovascular disease through a variety of ways, including inflammation, atherosclerosis, and apoptosis.^[39]^ Phytosphingosine is a bioactive lipid produced by the hydrolysis of SM, and has specific signaling capacities.^[40]^ Phytosphingosine is an important metabolite that can activate apoptosis involved in sphingolipid metabolism.^[41]^ The results showed that the levels of SM and phytosphingosine in the CHD + AF group were significantly higher than those in the NC group. Palmitic acid is one of the most abundant free fatty acids (FFAs) in plasma, accounting for 27% of total plasma FFA.^[42]^ Palmitic acid can activate caspase-3 and caspase-7 in cardiomyocytes, leading to cardiomyocyte apoptosis.^[43]^ It was also found that the levels of palmitic acid in the AF and AF + CHD groups were significantly higher than those in the CHD and NC groups. In addition, the reduced ability of β-oxidation for mitochondrial fatty acids and the accretion of fatty acids will lead to the accumulation of carnitine, a toxic lipid intermediate. Subsequently, the accumulation of long-chain acyl carnitine in the cytoplasm may cause membrane instability by inhibiting the exchange of sodium and calcium ions in the sarcolemma, leading to the development of arrhythmia.^[44]^ Our study showed that levels of L-acetylcarnitine in the AF and AF + CHD groups were significantly higher than those in the CHD and NC groups.

The increases of xanthine, hypoxanthine, and uric acid in plasma reflect the abnormal metabolism of xanthine and excessive accumulation of ROS.^[45]^ Levels of hypoxanthine and its metabolite inosine were elevated in the plasma of patients with AF combined with CHD.^[46]^ Previous clinical studies on acute and chronic heart failure found that the levels of glucose were significantly increased.^[47]^ This study also found that the levels of D-glucose in the CHD group were significantly higher than those in the NC group, but the levels of D-glucose in the AF and AF + CHD groups were significantly lower than those in the NC group. Amino acids play an important role in many biological processes of myocardial cells and participate in the development of a variety of cardiovascular diseases.^[48,49]^ The increase of branched chain amino acids (leucine, isoleucine, and valine) can promote arrhythmia.^[50]^ This study showed that the levels of L-valine in the AF + CHD group were significantly higher than that in the NC group, in line with previous results.

One notable observation from our data was that heightened serum levels of some oxylipins were observed in the AF + CHD group via our results. Analysis of enriched metabolic pathways led us to find that the significant metabolic alterations were mainly associated with α-linolenic acid, linoleic acid, arachidonic acid, and fatty acid metabolism, which were the most significant metabolic pathways in patients with AF and CHD. In recent years, it has been found that eicosanoids produced by PUFA metabolism play an important role in the occurrence and development of cardiovascular diseases including inflammation, arrhythmia, atherosclerosis, myocardial hypertrophy, thrombosis, and heart failure.^[51,52,53]^ Internal standard and multiple reaction monitoring (MRM) techniques were used to quantify 99 oxylipins in this study.

There were 18 particularly unique metabolites identified in samples from the AF + CHD group, including prostaglandins, leukotrienes, hydroxy-docosahexaenoic acids, hydroxy-eicostetraenoic acids, and hydroxy-eicosatrienoic acid. The results showed that the levels of PGD1, 6-keto-PGE1, PGJ2, 15-keto-PGD2, PGE3, and PGF3α were significantly increased in the AF + CHD group. Furthermore, the levels of PGE3, PGJ2, and 15-keto-PGD2 were positively associated with echocardiographic indicators of myocardial remodeling after adjusting for age, sex, smoking status, diabetes, hypertension, hyperlipidemia, heart failure, AF, and CHD. Prostaglandins are produced through the metabolism of dihomo-γ-linolenic acid/ arachidonic acid/eicosapentaenoic acid by the COX enzyme. COX-2 is the major source of prostaglandins in inflammatory conditions and diseases such as cardiovascular disease.^[54]^ The upregulation of COX-2 level was related to the release of atrial natriuretic peptide/brain natriuretic peptide and myocardial hypertrophy, and inhibition of COX-2 could reduce myocardial hypertrophy induced by angiotensin II.^[55,56]^ In the AF + CHD group, an increase in 12-oxo-LTB4 was observed in the study. 12-oxo-LTB4 is a key intermediate in LTB4 metabolism. Arachidonic acid is converted to LTB4 by 5-lipoxygenase. Given that LTB4 is a strong leukocyte chemoattractant and aggregator, and an important inflammatory mediator, inhibition of LTB4 signaling its receptor BLT1 could represent a pathway through which inflammation and apoptosis of cardiomyocytes is mediated in AF + CHD subjects.^[57]^

Inflammation is an important mechanism for the progression of coronary atherosclerotic plaques.^[58]^ The 12/15-LOX pathway in arachidonic acid metabolism is involved in the development of many inflammatory diseases, including cardiovascular disease, hypertension, asthma, and arthritis.^[59]^ Most HETEs produced by the LOX pathway are proinflammatory, and 12-HETE is involved in inducing cell hypertrophy and fibrosis,^[60]^ while 15-HETE is considered to be related to heart failure caused by cardiomyocyte fibrosis.^[61]^ It had been reported that eicosanoids can be used as non-invasive biomarkers of liver fibrosis. As biomarkers, 14-HDoHE and EPA were correlated with liver collagen content, and 16-HDoHE and tetranor 12-HETE were correlated with the stage of liver fibrosis.^[62]^ In this study, the levels of 16-HDoHE, 11-HETE, 12-HETE, 15-HETE, 12(S)-HpETE, tetranor 12-HETE, 14-HDoHE, 8-HDoHE, 15-HETrE, 5,15-diHETE, and 17 HDoHE were significantly increased in the AF + CHD group, indicating the presence of inflammatory response in patients, which was consistent with previously reported results.^[59,60]^ Additionally, 14-HDoHE levels were positively associated with echocardiographic indicators of myocardial remodeling in our results. These particular oxylipins could be of great significance for patients with AF with CHD, and may even be indicative of cases AF or of CHD developing a co-morbid condition with the corresponding disease state.

Specific oxylipins are not only associated with the progression of AF or CHD, but also have certain predictive value for disease prognosis. A study shows that the levels of six quantitative oxygen levels in plasma decrease with the increase of the number of coronary artery lesions; In high CHD risk symptomatic patients and ≥ 70% stenosis patients, those with higher 5-year survival rates have lower concentrations of five oxylipins and higher concentrations of one oxylipin; The panel composed of two types of oxylipins has high sensitivity in predicting survival during follow-up.^[63]^ However, this study was recently completed and long-term follow-up of patients was not conducted. In the future, we will obtain further evidence on the relationship between oxylipins and disease prognosis.

In this study, we used clinical heart failure as the diagnostic criteria for heart failure. The AF + CHD group and the control group had different incidences of heart failure, but the LVEF readings of the two groups were similar. The classification of heart failure in our study was based on the clinical diagnosis provided by the attending physicians, in accordance with guidelines such as those from the American Heart Association/American College of Cardiology.^[35]^ This diagnosis takes into account not only echocardiographic parameters like LVEF but also clinical symptoms (dyspnoea, fatigue, activity intolerance, and exercise limitation), signs of fluid overload, and elevated natriuretic peptide levels. The distributions of LVEF, NTproBNP, and renal function of the nine patients with heart failure are also shown in Table S8. It is important to note that heart failure can occur even when ejection fraction is preserved, a condition known as heart failure with preserved ejection fraction (HFpEF). AF is also a variable for scoring H2FPEF, which has been validated for the diagnosis of HFpEF.^[64]^ Furthermore, our study excluded patients with severe heart failure (NYHA ≥ III class), however we included patients with HFpEF, which could explain why the LVEF was preserved despite the presence of heart failure. In the future, more large-scale clinical studies will further clarify whether different types of heart failures may be diagnosable from their metabolic profiles.

Medications may affect changes in the patient’s metabolic pathways. In patients with ischemic heart disease and/or AF, α-linolenic acid-linoleic acid-arachidonic acid pathway is altered since both these pathologies are prothrombotic states, and platelet aggregation is mainly mediated by the products of cyclooxygenase.^[32]^ Aspirin, as a commonly used primary preventive drug for cardiovascular and cerebrovascular diseases, may change α-linolenic acid-linoleic acid-arachidonic acid pathway.^[33]^ In addition, thrombosis and inflammation are interdependent processes with the same mediators, including α-linolenic acid-linoleic acid-arachidonic acid pathway molecules,^[32]^ so the use of anticoagulant treatment can change these pathways. Some of the patients selected in this study are currently receiving aspirin treatment, which may alter the metabolic pathways mentioned above. Our research results also support this theory. We found that the level of PGD1, 12-oxo-LTB4, 12(S)-HpETE, and 15-HETrE were different between aspirin users and aspirin non-users. However, due to the insufficient number of enrolled cases in this study to support further analysis, we cannot elucidate the actual impact of drug therapy on metabolic pathways, which is one of the limitations of this study. The results of this study preliminarily suggest that the differential metabolites of patients with AF and CHD are similar to those of patients with AF only, but different from those of patients with CHD only, suggesting that the analysis of treatment is essential for understanding the results. In the future, large-scale clinical studies are needed to clarify the impact of aspirin and other drugs on the metabolic pathways of patients with AF and CHD.

This study identified the oxylipin signature in patients with AF and CHD, but five limitations are noted. Firstly, the underlying mechanisms through which the observed metabolic changes affect AF, CHD, and AF + CHD will require detailed exploration and larger independent data sets. Secondly, the biological functions of those key metabolites in AF + CHD patients need further confirmation. Thirdly, the effects of statins and other drugs on oxylipins should also be investigated in-depth in the future. Also, within the 1-year enrollment period, due to the limitations of enrollment conditions and testing costs, the number of cases included in our study is relatively small, and we could not conduct in-depth analysis for other clinical comorbidities, such as different types of heart failures. Finally, the distributions of some baseline clinical characteristics were slightly different among these four groups, which could have presented as confounds in the metabolomics analysis, and we exclude patients with some severe disease conditions, like congenital heart disease, valvular heart disease, severe heart failure, abnormal liver function, severe kidney disorders et.al. These might be the reasons why these patients were without high degree of AF or CHD. Future studies would involve recruitment of a more homogenous sample for each treatment group and patients with more severe conditions in the study design.

## Conclusion

A combination of untargeted and targeted metabolomics methods was successfully applied to derive molecular signatures for healthy controls, patients with AF, patients with CHD, and patients with both AF and CHD. This study reports that metabolic pathways for α-linolenic acid, linoleic acid, arachidonic acid, and fatty acid were the most significantly affected in patients with these heart diseases. Patients with AF co-morbid with CHD had a unique metabolite signature featuring multiple oxylipin species, and the presence of these oxylipins in serum samples was correlated with disease traits. Oxylipins could be potential intervention targets for AF or CHD, or the progression to a co-morbid disease.

## Supplementary Material

Supplementary Material
