# Supplementary material for: Integrated untargeted/targeted metabolomics identifies a putative oxylipin signature in patients with atrial fibrillation and coronary heart disease
Source: J Transl Int Med. 2024 Nov 6;12(5):495–509. doi: 10.1515/jtim-2023-0141 (PMC11538890; doi:10.1515/jtim-2023-0141)
Supplement: Supplementary file 1 — Supplementary Material [file jtim-2023-0141_sm.pdf]

# Integrated untargeted/targeted metabolomics identifies a putative oxylipin signature in patients with atrial fibrillation and coronary heart disease

**Lei Li[1†], Yingyuan Lu[2†], Zhiyong Du[3†], Meng Fang[2], Ying Wei[1], Wenxin Zhang[2], Yisheng Xu[4], Jiaxu Sun[2], Xiangrui Zeng[2], Guomin Hu[1], Lingli Wang[1], Yong Jiang[2], Shuwang Liu[1], Yida Tang[1], Haiyi Yu[1*], Pengfei Tu[2*] and Xiaoyu Guo[2*]**

[1]Department of Cardiology, Peking University Third Hospital; State Key Laboratory of Vascular Homeostasis and Remodeling, Peking University; NHC Key Laboratory of Cardiovascular Molecular Biology and Regulatory Peptides, Peking University; Beijing Key Laboratory of Cardiovascular Receptors Research, Beijing 100191, China

[2]State Key Laboratory of Natural and Biomimetic Drugs, School of Pharmaceutical Sciences, Peking University, Beijing 100191, China

[3]The Key Laboratory of Remodeling-Related Cardiovascular Diseases, Ministry of Education, National Clinical Research Center for Cardiovascular Diseases, Beijing Anzhen Hospital, Capital Medical University, Beijing 100029, China, Beijing Institute of Heart Lung and Blood Vessel Disease, Beijing 100029, China

[4]Waters Technologies Ltd., Beijing, 102600, China

[†]These authors contributed equally to this work.

*Correspondence: yuhaiyi@bjmu.edu.cn, pengfeitu@bjmu.edu.cn, and guoxiaoyu@bjmu.edu.cn

***Running head:*** *Metabolic insights in patients with AF and CHD*

**Table S1** Characteristic MRM transitions of 20 deuterated internal standards

| Compound | Time (min) | Q1 (Da) | Q3 (Da) | DP (V) | CE (eV) |
|---|---|---|---|---|---|
| 11,12-EET-$d_{11}$ | 5 | 330.2 | 179.1 | -80 | -18 |
| 14,15-EET-$d_{11}$ | 5 | 330.2 | 219 | -90 | -16 |
| 8,9-EET-$d_{11}$ | 5 | 330.3 | 154.8 | -85 | -18 |
| 12,13-diHOME-$d_4$ | 5 | 317.2 | 185.1 | -90 | -30 |
| 13-HODE-$d_4$ | 5 | 299.1 | 198 | -100 | -25 |
| 15d $PGJ_2$-$d_4$ | 5 | 319.1 | 275.2 | -90 | -20 |
| 6k $PGF_{1\alpha}$-$d_4$ | 5 | 373.2 | 167 | -100 | -35 |
| 8-iso $PGF_{2\alpha}$VI-$d_4$ | 5 | 364.2 | 115 | -100 | -30 |
| 9,10-diHOME-$d_4$ | 5 | 317.2 | 203 | -90 | -28 |
| 9-HODE-$d_4$ | 5 | 298.9 | 171.9 | -105 | -25 |
| dhk $PGF_{2\alpha}$-$d_4$ | 5 | 357.1 | 187.1 | -110 | -33 |
| $LTB_4$-$d_4$ | 5 | 339.1 | 197 | -95 | -23 |
| $PGD_2$-$d_4$ | 5 | 355.2 | 275.2 | -60 | -24 |
| $PGE_2$-$d_4$ | 5 | 355.21 | 275.4 | -50 | -23 |
| Resolvin $E_1$-$d_4$ | 5 | 353 | 197 | -100 | -22 |
| $TXB_2$-$d_4$ | 5 | 373.2 | 173.2 | -80 | -24 |
| 12-HETE-$d_8$ | 5 | 327.1 | 184.1 | -90 | -20 |
| 15-HETE-$d_8$ | 5 | 327.1 | 226.1 | -80 | -18 |
| 5-HETE-$d_8$ | 5 | 327.2 | 115.8 | -85 | -19 |
| Arachidonic acid-$d_8$ | 5 | 311.1 | 267.1 | -110 | -19 |

**Table S2** Characteristic MRM transitions of 99 oxylipins

| Compound | Time (min) | Q1 (Da) | Q3 (Da) | DP (V) | CE (eV) |
|---|---|---|---|---|---|
| 10(S),17(S)-DiHDoHE | 5 | 359.2 | 153.2 | -90 | -20 |
| 10-HDoHE | 5 | 343 | 153 | -85 | -19 |
| 11,12-DiHETrE | 5 | 337 | 167 | -90 | -30 |
| 11,12-EET | 5 | 319 | 167 | -90 | -20 |
| 11d-TXB2 | 5 | 367 | 305 | -50 | -23 |
| 11-HDoHE | 5 | 343 | 149 | -85 | -19 |
| 11-HETE | 5 | 319.001 | 167.001 | -85 | -19 |
| 11β-dhk-PGF2α | 5 | 333 | 221 | -110 | -19 |
| 11β-PGE2 | 5 | 351 | 271 | -95 | -23 |
| 12(S)-HpETE | 5 | 317.1 | 153 | -90 | -20 |
| 12,13-DiHODE | 5 | 311.2 | 293 | -50 | -29 |
| 12,13-DiHOME | 5 | 313 | 183 | -90 | -30 |
| 12,13-EpOME | 5 | 295 | 195 | -90 | -30 |
| 12-epi-LTB4 | 5 | 335.003 | 195.003 | -95 | -23 |
| 12-HEPE | 5 | 317 | 179 | -90 | -20 |
| 12-HETE | 5 | 319 | 135 | -90 | -16 |
| 12-HHTrE | 5 | 279 | 217 | -90 | -20 |
| 12-oxo-LTB4 | 5 | 335 | 253 | -95 | -23 |
| 12-oxoETE | 5 | 317 | 153 | -90 | -16 |

| | | | | | |
|---|---|---|---|---|---|
| 13,14-dihydro-15-keto-PGF1α | 5 | 355.2 | 193.2 | -100 | -26 |
| 13-HDoHE | 5 | 343 | 221 | -85 | -19 |
| 13-HODE | 5 | 295.001 | 195.001 | -105 | -25 |
| 13-HOTrE | 5 | 293 | 195 | -100 | -25 |
| 13-HOTrE(y) | 5 | 293 | 193 | -100 | -25 |
| 13-HpODE | 5 | 293.1 | 113 | -105 | -25 |
| 13-oxoODE | 5 | 293 | 167 | -105 | -25 |
| 14,15-DiHETrE | 5 | 337 | 207 | -90 | -28 |
| 14,15-EET | 5 | 319 | 175 | -80 | -18 |
| 14-HDoHE | 5 | 343 | 205 | -90 | -16 |
| 15d-PGD2 | 5 | 333.003 | 271.003 | -95 | -23 |
| 15-HEPE | 5 | 317 | 219 | -85 | -19 |
| 15-HETE | 5 | 319.001 | 175 | -85 | -19 |
| 15-HETrE | 5 | 321 | 221 | -85 | -19 |
| 15-HpETE | 5 | 317 | 113 | -85 | -19 |
| 15k-PGD2 | 5 | 349 | 235 | -95 | -23 |
| 15-oxoETE | 5 | 317 | 113 | -90 | -16 |
| 16-HDoHE | 5 | 343 | 233 | -85 | -19 |
| 16-HETE | 5 | 319 | 189 | -30 | -21 |
| 17 HDoHE | 5 | 343 | 229 | -110 | -19 |
| 17-HETE | 5 | 319 | 247 | -50 | -20 |

| | | | | | |
|---|---|---|---|---|---|
| 18-HETE | 5 | 319 | 261 | -60 | -20 |
| 19-OH-PGE2 | 5 | 367 | 243 | -50 | -23 |
| 2,3-dinor-11β-PGF2α | 5 | 325 | 227 | -100 | -26 |
| 2,3-dinor-6k-PGF1α | 5 | 363 | 281 | -95 | -23 |
| 20-COOH-AA | 5 | 333 | 271 | -50 | -23 |
| 20-COOH-LTB4 | 5 | 365 | 303 | -80 | -24 |
| 20-HETE | 5 | 319 | 245 | -85 | -18 |
| 5(S)-HpETE | 5 | 317.1 | 203.1 | -85 | -19 |
| 5,15-DiHETE | 5 | 335 | 201 | -95 | -23 |
| 5,6-DiHETrE | 5 | 337 | 145 | -90 | -28 |
| 5,6-EET | 5 | 319 | 191 | -85 | -19 |
| 5-HEPE | 5 | 317 | 115 | -85 | -19 |
| 5-HETE | 5 | 319 | 115 | -85 | -18 |
| 5-HETrE | 5 | 321 | 205 | -85 | -19 |
| 5-iso-PGF2α-VI | 5 | 353 | 115 | -100 | -26 |
| 5-oxoETE | 5 | 317 | 203 | -85 | -19 |
| 6k-PGE1 | 5 | 367 | 331 | -100 | -35 |
| 6t-LTB4 | 5 | 335.002 | 195.002 | -95 | -23 |
| 7-HDoHE | 5 | 343 | 141 | -85 | -19 |
| 8,9-DiHETrE | 5 | 337 | 127 | -80 | -24 |
| 8-HDoHE | 5 | 343 | 109 | -85 | -19 |

| | | | | | |
|---|---|---|---|---|---|
| 8-HEPE | 5 | 317 | 155 | -90 | -20 |
| 8-HETE | 5 | 319.001 | 155.001 | -85 | -19 |
| 8-HETrE | 5 | 321 | 157 | -100 | -26 |
| 8-iso-PGF2α-III | 5 | 353 | 193 | -110 | -33 |
| 8-iso-PGF3α | 5 | 351 | 307 | -30 | -28 |
| 9,10,13-TriHOME | 5 | 329.2 | 171.1 | -105 | -25 |
| 9,10-DiHOME | 5 | 313 | 201 | -90 | -28 |
| 9,10-EpOME | 5 | 295 | 171 | -90 | -28 |
| 9,12,13-TriHOME | 5 | 329.2 | 211.2 | -105 | -25 |
| 9-HODE | 5 | 295.001 | 171.001 | -105 | -25 |
| 9-HOTrE | 5 | 293 | 171 | -105 | -25 |
| 9-HpODE | 5 | 293.1 | 185 | -105 | -25 |
| 9-oxoODE | 5 | 293 | 185 | -105 | -25 |
| Adrenic acid | 5 | 331 | 287 | -110 | -33 |
| Arachidonic acid | 5 | 303 | 259 | -110 | -19 |
| dh-PGF2α | 5 | 355 | 283 | -100 | -26 |
| DHA | 5 | 327 | 283 | -50 | -23 |
| dhk-PGF2α | 5 | 353 | 291 | -60 | -24 |
| dihomo-15d-PGD2 | 5 | 361 | 299 | -50 | -23 |
| dihomo-PGF2α | 5 | 381 | 337 | -40 | -37 |
| EPA | 5 | 301 | 257 | -110 | -19 |

| | | | | | |
|---|---|---|---|---|---|
| HXA3 | 5 | 335 | 195 | -90 | -20 |
| HXB3 | 5 | 335 | 183 | -95 | -23 |
| LTB4 | 5 | 335.001 | 195.001 | -95 | -23 |
| PGA2 | 5 | 333.001 | 271.001 | -50 | -23 |
| PGB2 | 5 | 333.002 | 271.002 | -90 | -20 |
| PGD1 | 5 | 353 | 235 | -50 | -23 |
| PGD3 | 5 | 349 | 269 | -50 | -23 |
| PGE3 | 5 | 349.001 | 269.001 | -50 | -23 |
| PGEM | 5 | 327 | 291 | -60 | -24 |
| PGF1α | 5 | 355 | 293 | -95 | -23 |
| PGF3α | 5 | 351 | 193 | -50 | -23 |
| PGJ2 | 5 | 333 | 189 | -50 | -23 |
| PGK2 | 5 | 349 | 249 | -100 | -22 |
| Tetranor 12-HETE | 5 | 265 | 109 | -90 | -20 |
| Tetranor-PGDM | 5 | 327 | 247 | -100 | -26 |
| Tetranor-PGEM | 5 | 327.1 | 309.2 | -50 | -23 |
| Tetranor-PGFM | 5 | 329.2 | 311.2 | -100 | -26 |

**Table S3.** Associations between the differential oxylipins and clinical risk factors

| | **Male (*n* = 128)** | **Female (*n* = 31)** | |
|---|---|---|---|
| **Oxylipins** | **median [IQR]** | **median [IQR]** | ***P*** |

| | | | |
|---|---|---|---|
| PGD1 | 0.68 [0.42, 1.18] | 0.71 [0.49, 1.15] | 0.839 |
| 6-keto-PGE1 | 2.77 [1.15, 6.53] | 2.32 [0.96, 6.01] | 0.492 |
| PGE3 | 6.47 [5.17, 8.20] | 6.75 [5.57, 8.32] | 0.406 |
| PGF3α | 31.41 [17.41, 62.03] | 25.56 [16.31, 36.62] | 0.145 |
| PGJ2 | 1.54 [1.24, 2.07] | 1.70 [1.31, 2.03] | 0.59 |
| 15-keto-PGD2 | 0.22 [0.13, 0.32] | 0.23 [0.15, 0.29] | 0.976 |
| 12-oxo-LTB4 | 1225.66 [782.17, 1638.71] | 1376.81 [604.34, 1704.39] | 0.653 |
| 16-HDoHE | 10.19 [3.77, 21.83] | 9.86 [5.53, 21.07] | 0.688 |
| 11-HETE | 25.81 [8.59, 85.11] | 18.33 [9.77, 42.43] | 0.564 |
| 12-HETE | 40.15 [11.01, 132.29] | 31.51 [8.17, 171.26] | 0.941 |
| 15-HETE | 14.80 [8.32, 33.54] | 11.45 [8.03, 31.49] | 0.437 |
| 12(S)-HpETE | 9.75 [6.32, 14.01] | 13.08 [7.66, 17.36] | 0.039 |
| tetranor 12-HETE | 3.36 [1.74, 5.93] | 2.55 [1.87, 6.76] | 0.854 |
| 14-HDoHE | 34.00 [13.73, 88.21] | 44.86 [14.81, 100.46] | 0.575 |
| 8-HDoHE | 9.40 [4.71, 25.17] | 11.30 [4.51, 23.88] | 0.976 |
| 15-HETrE | 9.92 [6.14, 16.99] | 8.90 [5.22, 14.32] | 0.563 |
| 5,15-diHETE | 62.42 [52.79, 75.67] | 65.55 [58.87, 76.08] | 0.471 |
| 17 HDoHE | 2.75 [1.87, 3.98] | 2.01 [1.33, 3.11] | 0.035 |

| | **No Smoking (*n* = 81)** | **Smoking (*n* = 78)** | |
|---|---|---|---|
| **Oxylipins** | **median [IQR]** | **median [IQR]** | ***P*** |
| PGD1 | 0.79 [0.41, 1.23] | 0.64 [0.43, 1.03] | 0.496 |
| 6-keto-PGE1 | 2.40 [1.16, 5.36] | 3.04 [1.06, 7.96] | 0.448 |

| | | | |
|---|---|---|---|
| PGE3 | 6.83 [5.43, 8.22] | 6.43 [4.91, 8.56] | 0.711 |
| PGF3α | 28.68 [18.58, 47.70] | 32.76 [15.65, 70.71] | 0.518 |
| PGJ2 | 1.63 [1.33, 2.00] | 1.49 [1.18, 2.54] | 0.749 |
| 15-keto-PGD2 | 0.22 [0.14, 0.29] | 0.22 [0.12, 0.34] | 0.711 |
| 12-oxo-LTB4 | 1187.58 [732.19, 1598.75] | 1288.56 [776.04, 1756.29] | 0.332 |
| 16-HDoHE | 9.51 [4.51, 21.61] | 10.30 [4.61, 21.07] | 0.901 |
| 11-HETE | 23.89 [7.59, 81.78] | 29.30 [9.40, 83.12] | 0.373 |
| 12-HETE | 31.51 [11.02, 171.26] | 42.36 [10.57, 130.84] | 0.899 |
| 15-HETE | 14.52 [8.03, 37.38] | 15.07 [8.32, 32.26] | 0.934 |
| 12(S)-HpETE | 11.15 [6.94, 14.86] | 10.04 [6.32, 14.73] | 0.466 |
| tetranor 12-HETE | 2.51 [1.52, 5.40] | 3.50 [2.37, 6.16] | 0.107 |
| 14-HDoHE | 34.84 [13.68, 83.57] | 35.39 [14.81, 92.19] | 0.729 |
| 8-HDoHE | 8.22 [3.43, 23.31] | 11.87 [6.40, 27.68] | 0.136 |
| 15-HETrE | 8.97 [5.84, 15.67] | 10.22 [6.12, 17.92] | 0.503 |
| 5,15-diHETE | 60.85 [49.99, 73.99] | 64.24 [56.95, 75.78] | 0.117 |
| 17 HDoHE | 2.49 [1.73, 3.74] | 2.65 [1.66, 3.81] | 0.672 |

| | **No Diabetes (*n* = 124)** | **Diabetes (*n* = 35)** | |
|---|---|---|---|
| **Oxylipins** | **median [IQR]** | **median [IQR]** | ***P*** |
| PGD1 | 0.69 [0.42, 1.20] | 0.64 [0.45, 0.91] | 0.605 |
| 6-keto-PGE1 | 2.32 [1.07, 5.66] | 3.88 [1.43, 8.04] | 0.11 |
| PGE3 | 6.48 [5.17, 8.20] | 7.08 [5.20, 8.62] | 0.369 |
| PGF3α | 28.88 [16.02, 49.32] | 33.89 [25.41, 71.46] | 0.122 |

| | | | |
|---|---|---|---|
| PGJ2 | 1.59 [1.29, 2.03] | 1.60 [1.22, 2.42] | 0.832 |
| 15-keto-PGD2 | 0.22 [0.13, 0.29] | 0.22 [0.13, 0.35] | 0.722 |
| 12-oxo-LTB4 | 1154.47 [714.97, 1636.59] | 1368.46 [934.06, 1773.23] | 0.171 |
| 16-HDoHE | 10.54 [3.74, 21.10] | 10.07 [6.01, 26.38] | 0.443 |
| 11-HETE | 25.37 [9.03, 79.57] | 23.89 [7.72, 85.11] | 0.857 |
| 12-HETE | 40.15 [12.10, 134.94] | 27.92 [7.94, 164.23] | 0.694 |
| 15-HETE | 14.14 [8.32, 33.37] | 19.42 [7.73, 35.12] | 0.794 |
| 12(S)-HpETE | 9.93 [6.37, 14.01] | 11.43 [7.08, 15.67] | 0.323 |
| tetranor 12-HETE | 3.10 [1.58, 5.53] | 3.55 [2.53, 7.81] | 0.109 |
| 14-HDoHE | 35.51 [13.09, 86.90] | 34.00 [21.41, 96.57] | 0.33 |
| 8-HDoHE | 9.08 [4.64, 23.68] | 14.12 [4.70, 27.73] | 0.424 |
| 15-HETrE | 9.75 [6.11, 15.16] | 11.78 [6.26, 18.54] | 0.482 |
| 5,15-diHETE | 62.90 [54.76, 75.52] | 61.43 [50.71, 78.11] | 0.672 |
| 17 HDoHE | 2.67 [1.66, 3.80] | 2.46 [1.89, 3.81] | 0.73 |
| | **No Hypertension (*n* = 70)** | **Hypertension (*n* = 89)** | |
| **Oxylipins** | **median [IQR]** | **median [IQR]** | ***P*** |
| PGD1 | 0.65 [0.42, 1.04] | 0.69 [0.42, 1.21] | 0.427 |
| 6-keto-PGE1 | 2.48 [1.15, 6.46] | 2.87 [0.95, 5.86] | 0.887 |
| PGE3 | 6.72 [5.20, 8.22] | 6.51 [5.18, 8.22] | 0.871 |
| PGF3α | 27.92 [15.49, 47.10] | 31.74 [21.87, 63.63] | 0.105 |
| PGJ2 | 1.64 [1.31, 2.01] | 1.49 [1.24, 2.17] | 0.716 |
| 15-keto-PGD2 | 0.24 [0.14, 0.31] | 0.21 [0.12, 0.31] | 0.417 |

| | | | |
|---|---|---|---|
| 12-oxo-LTB4 | 1369.51 [893.88, 1635.88] | 1121.90 [685.92, 1699.87] | 0.266 |
| 16-HDoHE | 11.57 [4.37, 21.96] | 9.70 [4.63, 21.03] | 0.585 |
| 11-HETE | 35.82 [10.45, 87.03] | 19.31 [8.21, 74.61] | 0.148 |
| 12-HETE | 44.16 [16.69, 151.49] | 30.34 [8.40, 122.77] | 0.183 |
| 15-HETE | 18.37 [8.98, 35.75] | 13.55 [7.12, 30.92] | 0.17 |
| 12(S)-HpETE | 11.15 [7.39, 13.89] | 9.93 [5.81, 15.76] | 0.371 |
| tetranor 12-HETE | 3.26 [1.63, 6.05] | 3.34 [1.87, 5.77] | 0.741 |
| 14-HDoHE | 41.13 [13.68, 85.37] | 31.57 [14.25, 91.69] | 0.474 |
| 8-HDoHE | 10.29 [4.85, 26.83] | 10.14 [4.60, 23.58] | 0.823 |
| 15-HETrE | 10.43 [7.57, 16.46] | 9.51 [5.43, 16.88] | 0.384 |
| 5,15-diHETE | 64.24 [55.51, 76.79] | 61.11 [50.62, 74.32] | 0.283 |
| 17 HDoHE | 2.83 [1.90, 3.97] | 2.40 [1.52, 3.62] | 0.165 |

| **Oxylipins** | **No Hyperlipidemia (*n* = 100) median [IQR]** | **Hyperlipidemia (*n* = 59) median [IQR]** | ***P*** |
|---|---|---|---|
| PGD1 | 0.60 [0.41, 1.04] | 0.81 [0.49, 1.21] | 0.083 |
| 6-keto-PGE1 | 2.18 [1.07, 4.60] | 4.59 [1.10, 8.06] | 0.069 |
| PGE3 | 6.55 [5.17, 8.13] | 6.61 [5.24, 8.64] | 0.414 |
| PGF3α | 28.72 [15.53, 48.07] | 38.53 [25.65, 85.61] | 0.011 |
| PGJ2 | 1.62 [1.31, 2.01] | 1.54 [1.05, 2.31] | 0.813 |
| 15-keto-PGD2 | 0.22 [0.13, 0.30] | 0.23 [0.12, 0.33] | 0.923 |
| 12-oxo-LTB4 | 1159.44 [731.43, 1612.90] | 1422.65 [799.50, 1771.65] | 0.176 |
| 16-HDoHE | 10.10 [4.51, 22.26] | 10.19 [4.69, 20.24] | 0.88 |

| | | | |
|---|---|---|---|
| 11-HETE | 33.73 [11.92, 83.27] | 12.51 [5.52, 49.78] | 0.018 |
| 12-HETE | 52.34 [14.49, 148.60] | 28.47 [7.83, 91.17] | 0.11 |
| 15-HETE | 16.86 [8.81, 36.49] | 11.34 [6.79, 28.70] | 0.08 |
| 12(S)-HpETE | 10.63 [6.94, 14.47] | 10.35 [6.31, 15.94] | 0.949 |
| tetranor 12-HETE | 3.50 [1.89, 5.78] | 2.89 [1.83, 5.93] | 0.579 |
| 14-HDoHE | 37.88 [14.34, 92.93] | 32.31 [14.09, 87.03] | 0.585 |
| 8-HDoHE | 10.82 [5.66, 24.20] | 9.81 [3.59, 24.18] | 0.638 |
| 15-HETrE | 10.43 [6.84, 16.86] | 8.97 [4.71, 17.32] | 0.184 |
| 5,15-diHETE | 62.47 [52.60, 76.07] | 64.24 [55.98, 74.52] | 0.924 |
| 17 HDoHE | 2.47 [1.66, 3.60] | 2.78 [1.73, 4.04] | 0.343 |

| | **No Heart Failure (*n* = 150)** | **Heart Failure (*n* = 9)** | |
|---|---|---|---|
| **Oxylipins** | **median [IQR]** | **median [IQR]** | ***P*** |
| PGD1 | 0.68 [0.42, 1.19] | 0.80 [0.49, 0.91] | 0.89 |
| 6-keto-PGE1 | 2.72 [1.07, 6.09] | 4.50 [1.48, 7.87] | 0.362 |
| PGE3 | 6.45 [5.13, 8.17] | 8.12 [7.07, 8.63] | 0.094 |
| PGF3α | 28.97 [16.39, 49.86] | 94.71 [29.13, 183.03] | 0.088 |
| PGJ2 | 1.58 [1.25, 2.06] | 1.72 [1.33, 2.97] | 0.312 |
| 15-keto-PGD2 | 0.22 [0.13, 0.30] | 0.33 [0.22, 0.50] | 0.128 |
| 12-oxo-LTB4 | 1193.97 [747.52, 1635.88] | 1791.77 [1367.42, 1854.84] | 0.046 |
| 16-HDoHE | 10.19 [4.70, 21.07] | 12.13 [2.64, 46.22] | 0.812 |
| 11-HETE | 24.87 [8.54, 82.59] | 25.92 [10.36, 42.23] | 0.915 |
| 12-HETE | 37.15 [11.40, 134.94] | 22.66 [2.80, 164.23] | 0.583 |

| | | | |
|---|---|---|---|
| 15-HETE | 14.34 [8.31, 33.45] | 15.10 [9.21, 30.44] | 0.887 |
| 12(S)-HpETE | 10.52 [6.50, 14.71] | 11.43 [6.94, 14.96] | 0.92 |
| tetranor 12-HETE | 3.33 [1.85, 5.72] | 3.48 [1.40, 8.92] | 0.779 |
| 14-HDoHE | 35.51 [14.96, 86.99] | 14.25 [5.29, 140.61] | 0.77 |
| 8-HDoHE | 10.14 [4.95, 24.03] | 11.27 [2.98, 57.05] | 0.833 |
| 15-HETrE | 9.80 [6.11, 16.79] | 11.10 [7.40, 18.71] | 0.648 |
| 5,15-diHETE | 62.50 [53.06, 75.69] | 64.05 [58.49, 69.35] | 0.696 |
| 17 HDoHE | 2.60 [1.65, 3.82] | 2.46 [2.04, 3.07] | 0.952 |

**Table S4.** Correlations between the differential oxylipins and blood lipid indicators

| | Total Cholesterol | | LDL-C | |
|---|---|---|---|---|
| **Oxylipins** | **R** | ***P*** | **R** | ***P*** |
| PGD1 | -0.099 | 0.291 | -0.146 | 0.119 |
| 6-keto-PGE1 | -0.033 | 0.724 | -0.110 | 0.243 |
| PGE3 | -0.156 | 0.101 | -0.153 | 0.108 |
| PGF3α | 0.041 | 0.703 | 0.026 | 0.807 |
| PGJ2 | 0.059 | 0.535 | 0.023 | 0.805 |
| 15-keto-PGD2 | -0.051 | 0.590 | -0.046 | 0.626 |
| 12-oxo-LTB4 | 0.036 | 0.705 | 0.069 | 0.466 |
| 16-HDoHE | -0.057 | 0.552 | -0.035 | 0.718 |
| 11-HETE | 0.030 | 0.750 | 0.045 | 0.625 |
| 12-HETE | -0.104 | 0.284 | -0.114 | 0.240 |
| 15-HETE | -0.045 | 0.629 | -0.006 | 0.949 |
| 12(S)-HpETE | -0.040 | 0.667 | 0.003 | 0.972 |

| | | | | |
|---|---|---|---|---|
| tetranor 12-HETE | 0.089 | 0.347 | 0.075 | 0.431 |
| 14-HDoHE | -0.074 | 0.426 | -0.059 | 0.525 |
| 8-HDoHE | -0.045 | 0.644 | -0.050 | 0.604 |
| 15-HETrE | -0.045 | 0.633 | -0.013 | 0.892 |
| 5,15-diHETE | 0.079 | 0.406 | 0.039 | 0.679 |
| 17 HDoHE | -0.090 | 0.331 | -0.147 | 0.111 |

**Table S5.** The effect of aspirin on oxylipins levels

| | **No Aspirin (*n* = 94)** | **Aspirin (*n* = 64)** | |
|---|---|---|---|
| **Oxylipins** | **median [IQR]** | **median [IQR]** | ***P*** |
| PGD1 | 0.62 [0.38, 1.04] | 0.80 [0.49, 1.21] | 0.024 |
| 6-keto-PGE1 | 2.18 [1.07, 5.24] | 4.01 [1.19, 7.59] | 0.172 |
| PGE3 | 6.89 [5.25, 8.52] | 6.25 [4.91, 7.86] | 0.21 |
| PGF3α | 29.69 [16.10, 50.15] | 28.97 [17.61, 53.63] | 0.832 |
| PGJ2 | 1.62 [1.31, 2.01] | 1.51 [1.15, 2.34] | 0.716 |
| 15-keto-PGD2 | 0.24 [0.12, 0.33] | 0.19 [0.13, 0.29] | 0.301 |
| 12-oxo-LTB4 | 1306.64 [905.60, 1719.96] | 1016.82 [523.72, 1601.59] | 0.016 |
| 16-HDoHE | 10.30 [2.73, 21.06] | 9.41 [4.96, 22.37] | 0.633 |
| 11-HETE | 35.78 [18.62, 87.24] | 8.68 [4.78, 39.50] | <0.001 |
| 12-HETE | 45.69 [13.45, 148.74] | 28.29 [8.54, 93.23] | 0.329 |
| 15-HETE | 18.47 [9.29, 35.48] | 10.47 [6.12, 27.27] | 0.015 |
| 12(S)-HpETE | 11.27 [7.79, 15.65] | 7.39 [4.68, 13.15] | 0.007 |
| tetranor 12-HETE | 3.70 [2.03, 5.85] | 2.84 [1.53, 5.83] | 0.298 |
| 14-HDoHE | 35.78 [13.07, 92.10] | 33.05 [16.28, 85.93] | 0.982 |

| | | | |
|---|---|---|---|
| 8-HDoHE | 10.22 [4.24, 27.71] | 9.49 [4.95, 22.44] | 0.971 |
| 15-HETrE | 10.46 [7.43, 18.28] | 7.46 [4.55, 15.19] | 0.019 |
| 5,15-diHETE | 62.50 [54.24, 76.16] | 63.94 [54.11, 74.60] | 1 |
| 17 HDoHE | 2.29 [1.49, 3.78] | 2.78 [1.91, 4.00] | 0.209 |

**Table S6.** The effect of warfarin or direct oral anticoagulants on oxylipins levels

| | **No Warfarin or Direct Oral Anticoagulants (*n* = 58)** | **Warfarin or Direct Oral Anticoagulants (*n* = 69)** | |
|---|---|---|---|
| **Oxylipins** | **median [IQR]** | **median [IQR]** | ***P*** |
| PGD1 | 0.85 [0.53, 1.33] | 0.51 [0.33, 0.82] | <0.001 |
| 6-keto-PGE1 | 2.76 [1.45, 5.65] | 2.18 [1.02, 6.26] | 0.53 |
| PGE3 | 6.15 [4.80, 7.82] | 7.07 [5.75, 8.51] | 0.048 |
| PGF3α | 26.05 [15.89, 35.40] | 45.09 [24.90, 91.24] | 0.004 |
| PGJ2 | 1.52 [1.11, 2.00] | 1.62 [1.33, 2.08] | 0.121 |
| 15-keto-PGD2 | 0.21 [0.13, 0.28] | 0.25 [0.13, 0.35] | 0.29 |
| 12-oxo-LTB4 | 1066.10 [646.49, 1437.27] | 1520.62 [1029.29, 1811.32] | <0.001 |
| 16-HDoHE | 9.13 [4.10, 17.23] | 14.39 [2.90, 31.17] | 0.151 |
| 11-HETE | 16.14 [6.64, 35.82] | 41.71 [21.27, 100.03] | <0.001 |
| 12-HETE | 30.78 [13.04, 124.11] | 67.22 [17.44, 190.63] | 0.05 |
| 15-HETE | 12.67 [8.47, 24.41] | 23.30 [10.82, 41.24] | 0.004 |
| 12(S)-HpETE | 9.32 [5.72, 13.17] | 11.85 [8.16, 16.62] | 0.006 |
| tetranor 12-HETE | 2.67 [1.44, 4.25] | 4.09 [2.31, 7.64] | 0.001 |
| 14-HDoHE | 31.57 [13.09, 58.37] | 44.36 [14.12, 141.29] | 0.055 |
| 8-HDoHE | 9.02 [4.91, 18.90] | 15.84 [5.56, 41.68] | 0.036 |

| | | | |
|---|---|---|---|
| 15-HETrE | 7.76 [4.99, 10.69] | 13.28 [8.07, 20.40] | <0.001 |
| 5,15-diHETE | 64.24 [55.96, 77.24] | 60.85 [51.03, 72.38] | 0.128 |
| 17 HDoHE | 3.03 [1.89, 4.02] | 2.20 [1.48, 3.14] | 0.029 |

**Table S7.** The effect of statins on oxylipins levels

| | **No Statin ($n$ = 55)** | **Statin ($n$ = 103)** | |
|---|---|---|---|
| **Oxylipins** | **median [IQR]** | **median [IQR]** | ***P*** |
| PGD1 | 0.66 [0.43, 1.03] | 0.69 [0.42, 1.21] | 0.503 |
| 6-keto-PGE1 | 2.03 [1.07, 4.99] | 3.13 [1.07, 7.11] | 0.181 |
| PGE3 | 6.69 [5.05, 8.15] | 6.53 [5.21, 8.29] | 0.821 |
| PGF3α | 34.01 [15.50, 48.45] | 28.89 [18.55, 64.59] | 0.836 |
| PGJ2 | 1.63 [1.33, 2.00] | 1.50 [1.20, 2.18] | 0.491 |
| 15-keto-PGD2 | 0.23 [0.14, 0.27] | 0.22 [0.12, 0.33] | 0.948 |
| 12-oxo-LTB4 | 1207.73 [871.31, 1566.39] | 1264.71 [686.21, 1749.17] | 0.833 |
| 16-HDoHE | 10.85 [4.09, 20.16] | 9.99 [4.51, 21.99] | 0.971 |
| 11-HETE | 35.86 [21.61, 85.62] | 16.03 [7.02, 76.20] | 0.007 |
| 12-HETE | 53.29 [19.37, 151.10] | 28.70 [8.09, 111.70] | 0.053 |
| 15-HETE | 20.74 [9.29, 34.66] | 13.55 [7.21, 33.15] | 0.218 |
| 12(S)-HpETE | 11.56 [8.16, 14.29] | 9.77 [5.82, 15.32] | 0.163 |
| tetranor 12-HETE | 3.52 [2.02, 4.91] | 3.18 [1.54, 6.16] | 0.644 |
| 14-HDoHE | 38.11 [15.26, 78.75] | 33.62 [13.48, 93.14] | 0.596 |
| 8-HDoHE | 10.03 [6.77, 25.87] | 10.14 [4.08, 24.34] | 0.832 |
| 15-HETrE | 10.33 [7.11, 14.75] | 9.75 [5.23, 16.88] | 0.361 |

| | | | |
|---|---|---|---|
| 5,15-diHETE | 62.50 [57.23, 72.25] | 64.04 [50.38, 76.80] | 0.697 |
| 17 HDoHE | 2.48 [1.52, 3.58] | 2.66 [1.72, 3.98] | 0.427 |

**Table S8.** The distributions of LVEF, NTproBNP and renal function of the nine patients with heart failure

| **Group** | **NTproBNP (pg/mL)** | **Cr (μmol/L)** | **LVEF (%)** |
|---|---|---|---|
| AF+CHD | 131.0 | 89 | 78 |
| AF+CHD | 210.0 | 64 | 52 |
| AF+CHD | 3427.0 | 82 | 66 |
| AF+CHD | 929.2 | 85 | 52 |
| AF+CHD | 2813.0 | 103 | 47 |
| AF+CHD | 1745.0 | 77 | 68 |
| AF | 1876.0 | 81 | 67 |
| AF | 1665.0 | 67 | 66 |
| AF | 335.9 | 87 | 49 |

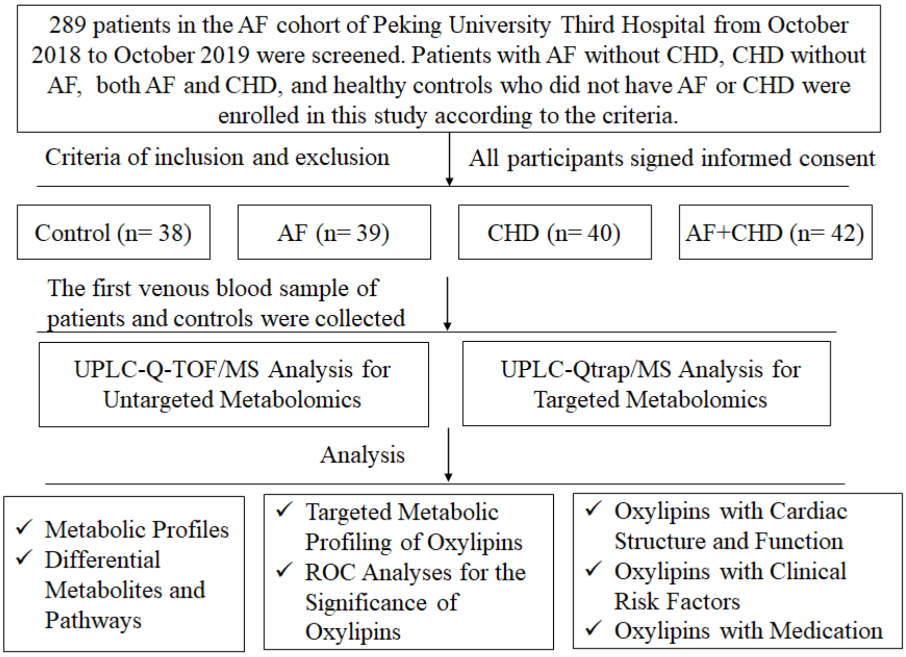


**Figure S1** The workflow of this study.

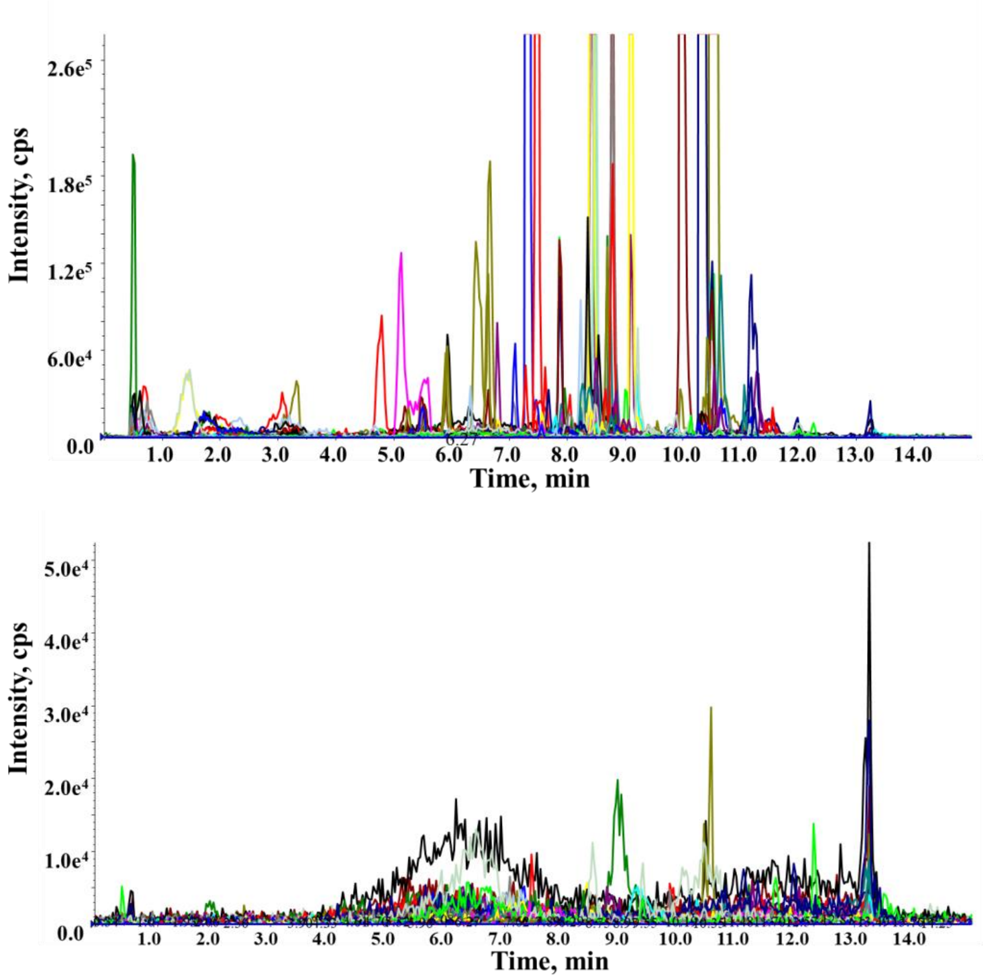


**Figure S2** The representative total ion chromatography chromatogram of oxylipins in serum samples

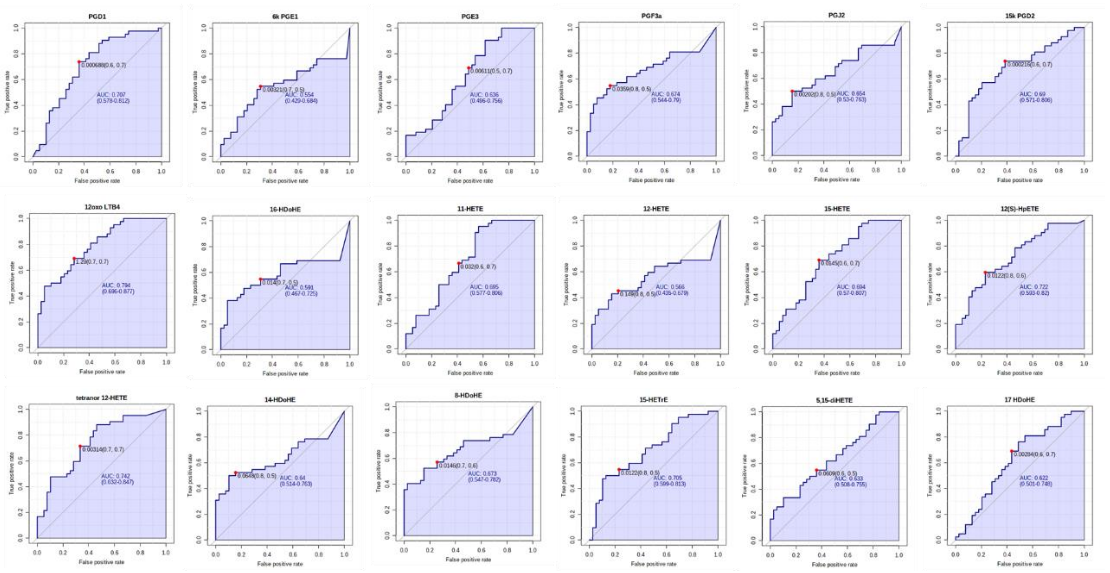


**Figure S3** Univariate ROC curve analysis for individual metabolic biomarkers of the AF + CHD group

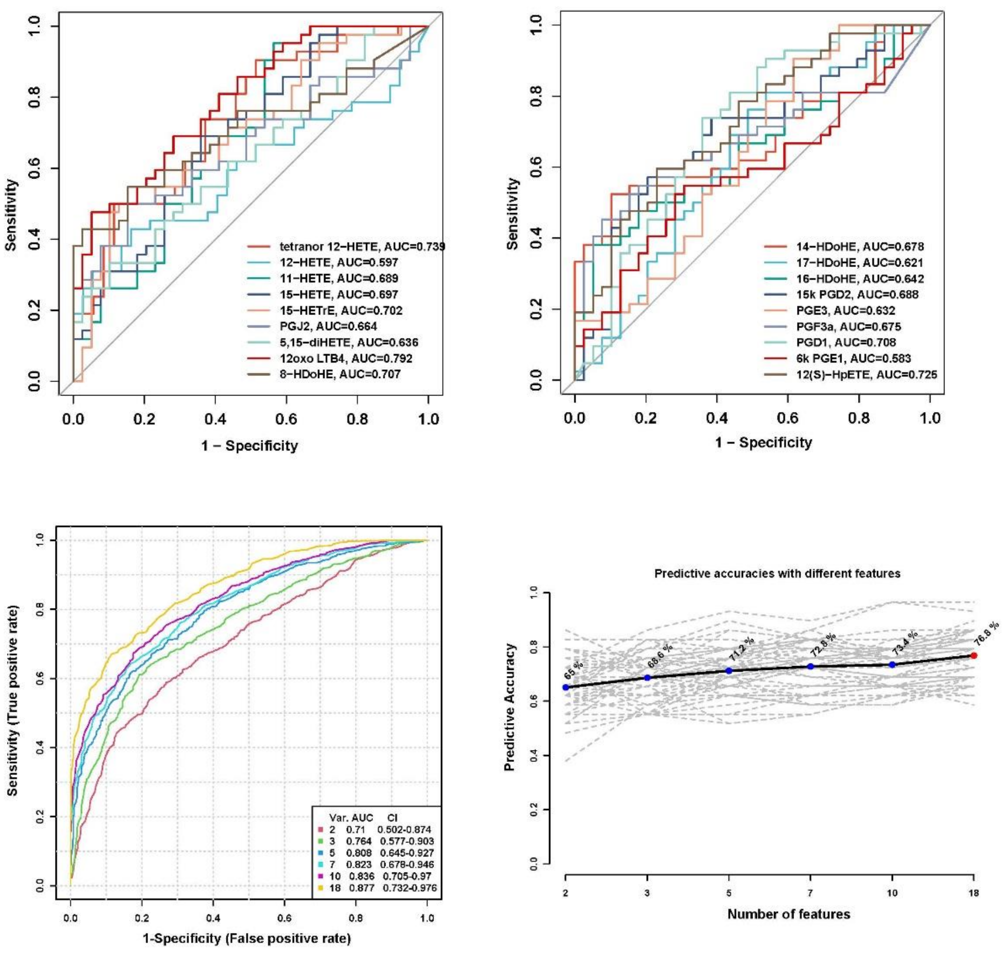


**Figure S4** ROC curve analysis for individual metabolic biomarkers of the AF + CHD group. Logistic regression-based ROC analyses of the diagnostic performances for the nine metabolite markers (A), the other nine metabolite markers (B); Random forest-based receiver operating characteristic curve analyses of the diagnostic performances for multiple metabolite combinations (C); Predictive accuracies with different features (D).
